# The Transcription Factor Sp3 Regulates the Expression of a Metastasis-Related Marker of Sarcoma, Actin Filament-Associated Protein 1-Like 1 (AFAP1L1)

**DOI:** 10.1371/journal.pone.0049709

**Published:** 2013-01-09

**Authors:** Yoichiro Kajita, Tomohisa Kato, Sakura Tamaki, Moritoshi Furu, Ryo Takahashi, Satoshi Nagayama, Tomoki Aoyama, Hiroyuki Nishiyama, Eijiro Nakamura, Toyomasa Katagiri, Yusuke Nakamura, Osamu Ogawa, Junya Toguchida

**Affiliations:** 1 Department of Tissue Regeneration, Institute for Frontier Medical Sciences, Kyoto University, Kyoto, Japan; 2 Department of Urology, Graduate School of Medicine, Kyoto University, Kyoto, Japan; 3 Department of Orthopaedic Surgery, Graduate School of Medicine, Kyoto University, Kyoto, Japan; 4 Department of Surgery, Graduate School of Medicine, Kyoto University, Kyoto, Japan; 5 Laboratory of Molecular Medicine, Human Genome Center, Institute of Medical Science, The University of Tokyo, Tokyo, Japan; 6 Center for iPS Cell Research and Application, Kyoto University, Kyoto, Japan; Peking University Health Science Center, China

## Abstract

We previously identified actin filament-associated protein 1-like 1 (AFAP1L1) as a metastasis-predicting marker from the gene-expression profiles of 65 spindle cell sarcomas, and demonstrated the up-regulation of *AFAP1L1* expression to be an independent risk factor for distant metastasis in multivariate analyses. Little is known, however, about how the expression of *AFAP1L1* is regulated. Luciferase reporter assays showed tandem binding motives of a specificity protein (Sp) located at −85 to −75 relative to the transcriptional start site to be essential to the promoter activity. Overexpression of Sp1 and Sp3 proteins transactivated the proximal *AFAP1L1* promoter construct, and electrophoretic mobility shift assays showed that both Sp1 and Sp3 were able to bind to this region *in vitro*. Chromatin immunoprecipitation experiments, however, revealed that Sp3 is the major factor binding to the proximal promoter region of the *AFAP1L1* gene in AFAP1L1- positive cells. Treatment with mithramycin A, an inhibitor of proteins binding to GC-rich regions, prevented Sp3 from binding to the proximal promoter region of *AFAP1L1* and decreased its expression in a dose-dependent manner. Finally, knocking down Sp3 using small inhibitory RNA duplex (siRNA) reduced AFAP1L1 expression significantly, which was partially restored by expressing siRNA-resistant Sp3. These findings indicate a novel role for Sp3 in sarcomas as a driver for expression of the metastasis-related gene *AFAP1L1*.

## Introduction

Soft tissue sarcoma (STS) is a malignant neoplasm that can arise in fat, muscle, fibrous tissue, blood vessels, or other supporting tissue in any part of the body. STSs are divided into two groups based on morphology; small round cell sarcomas and spindle cell sarcomas. The former include rhabdomyosarcomas and extraskeletal Ewing's tumors, against which chemotherapy and radiotherapy are effective at least in the initial stages, and therefore treatment other than surgery is usually the first choice. STSs in the latter group, such as leiomyosarcomas and malignant fibrous histiocytomas, however, are radio- and chemoresistant in most cases and therefore wide resection with proper surgical margins is the only way to control local tumors. In spite of proper treatment for local disease, approximately half of patients develop metastasis in distant organs, particularly in the lungs. Although recent studies have demonstrated a beneficial effect of chemotherapy, the improvement is far from satisfactory. Considering the associated side effects, it is desirable to identify high-risk patients, to whom additional treatments should be administered.

AFAP1L1 was previously identified as a metastasis-predicting marker from the gene-expression profiles of 65 spindle cell sarcomas by our group [1]. In univariate and multivariate analyses, higher expression of AFAP1L1 was found to contribute to the occurrence of distant metastases, along with patient age and tumor grade. Knocking down of the *AFAP1L1* gene in sarcoma cells reduced cell invasiveness and forced expression of *AFAP1L1* in immortalized human mesenchymal stem cells increased anchorage-independent cell growth as well as cell invasiveness. These results suggest that the molecular mechanism up-regulating the expression of *AFAP1L1* is a key to the progression of sarcomas. In this study, we explored the transcriptional regulation of *AFAP1L1* in order to find factors responsible for the up-regulation of AFAP1L1 expression, which will help us to understand how sarcoma cells gain the malignant phenotype.

## Materials and Methods

### Cell Lines, antibodies and reagents

Human osteosarcoma cell lines (U2OS, MG63, and Saos2) and a human fibrosarcoma cell line (HT1080) were obtained from American Type Culture Collection (ATCC, Manassas, VA). PC-3 (human prostate cancer) and 293T were also obtained from ATCC. SYO-1 (human synovial sarcoma cell line) [2] was provided by Dr. A. Kawai (National Cancer Center, Japan), and 293T was described elsewhere [3]. Informed consent was obtained from the patient with written consent, and the procedure was approved by the Ethics Committee of Graduate School of Medicine and Dentistry, Okayama University. Cells were cultured in DMEM (for U2OS, MG63, Saos2, 293T, HT1080 and SYO-1) or RPMI (for PC-3) supplemented with 10% fetal bovine serum, 0.1 mg/ml streptomycin, and 100 units/ml penicillin under 5% CO2 at 37°C. The anti-AFAP1L1 polyclonal antibody was produced in our laboratory as described previously [1]. The anti-Sp1 antibodies (1C6 and PEP2) and anti-Sp3 antibody (D-20) were purchased from Santa Cruz Biotechnology (Santa Cruz, CA). The anti-β-tubulin antibody was obtained from Thermo Fisher Scientific Inc. (Waltham, MA), and anti-acetylated H3K9 (06-942), from Millipore Corp (Billerica, MA). The anti-Flag antibody and mithramycin A were purchased from Sigma-Aldrich (St. Louis, MO).

### Semiquantitative reverse-transcription (RT)-PCR and quantitative real-time RT-PCR (qPCR)

The procedures for extracting total RNA and RT-PCR have been described previously [4]. Sets of primers for RT-PCR and qPCR are listed in Table S1. To quantitate AFAP1L1 expression, qPCR was performed in triplicate using TaqMan Universal Master Mix (Applied Biosystems, Foster City, CA) and a thermal cycler (ABI 7300 Real-Time PCR System, Applied Biosystems). qPCR for ChIP assays was done using SYBR GREEN reagent (Applied Biosystems) and a set of primers used in RT-PCR. Conditions for PCR and qPCR are available upon request.

### Plasmid constructs

Information on the 5′ flanking regulatory region of the *AFAP1L1* gene was obtained from GenBank (NC_000005.9). A 2,325-bp DNA fragment from −2250 to +75 relative to the transcription start site (TSS) was amplified by PCR using a sense primer with a XhoI site and an antisense primer with a HindIII site. DNA synthesis was performed with Primestar DNA polymerase (Takara, Shiga, Japan). The product was digested by XhoI and HindIII and cloned into a luciferase reporter plasmid, PGV-basic (Toyo Ink, Tokyo, Japan), to obtain PGV-(−2250). Other reporter vectors harboring a shorter DNA fragment (−1039, −778, −688, −601, −410, −224, −71, −53 or −46 to +75) were generated by a PCR-based method using PGV-(−2250) as a template. The primers used to amplify each fragment are listed in Table S1. Plasmids harboring mutations in the Sp-binding site (SBS) or Ets-binding site (EBS) were created by PCR-based mutagenesis using PGV-(−224) as a template. Briefly, PCR was performed with pairs of primers containing mutations in SBS1 (−86 -GGGCGGGGCGG- −76 to G*TT*CGG*TT*CGG), SBS2 (−102 -GGGCGG- −97 to G*TT*CGG), EBS1 (−60 -ATCCT- −56 to AT*AA*T) and EBS2 (−121 -TTCCG- −117 to TT*AA*G). The PCR product was digested by DpnI (TOYOBO, Osaka, Japan), transformed to competent cells and propagated. pEVR2/Sp1 and pRC/Sp3 were kindly provided by Dr. G. Suske (Marburg University, Marburg, Germany). Because pRC/Sp3 lacks the N-terminal part of the *Sp3* gene [5] , a vector that includes a full-length version of the *Sp3* gene was created as described previously [4]. Briefly, a PCR-amplified EcoRI-NotI fragment of the N-terminal part of *Sp3* and a NotI-XhoI fragment from pRC/Sp3 were sequentially cloned into pcDNA3.1(+) (Invitrogen, Carlsbad, CA), yielding pcDNA/Sp3(li-1), which contained a long isoform of the *Sp3* gene [5]. Using this vector as a template, another type of long isoform (li-2) [5] and two types of short isoform (si-1 and si-2) [5] were created by a PCR-based method and subcloned into pcDNA3.1(+) vectors, yielding pcDNA/Sp3(li-2), pcDNA/Sp3(si-1), and pcDNA/Sp3(si-2). Sequences of all the cDNAs were confirmed by sequencing. Plasmid vectors for Ets1, Ets2, ELK1, SAP1, PEA3 and dominant negative Ets (DN-Ets) were kindly provided by Dr. E. Hara (The Cancer Institute of Japanese Foundation for Cancer Research, Tokyo, Japan).

### Luciferase assays

Cells (2×10^4^) in 24-well dishes were transfected with 0.5 µg of each reporter plasmid and 2 ng of pRL-TK control vector (Toyo Ink) using Lipofectamine LTX (Invitrogen) according to the manufacturer's instructions. In the co-transfection experiments, the total amount of plasmid was adjusted with pcDNA3.1(+) to 2 µg. Cells were harvested at 24 h after transfection and luciferase assays were performed with the Dual Luciferase Assay Reporter System (Promega, Madison, WI) as described previously [4].

### Electrophoretic Mobility Shift Assay (EMSA)

Single-stranded oligonucleotides (ONDs) corresponding to sense and antisense sequences of the wild-type or mutated SBS1 site were synthesized (Table S1), and mutated ONDs (25 pmol) were end-labeled at 37°C for 30 min in a 50-µl reaction mixture containing 1 µl of [γ-^32^P]ATP and 10 units of T4 Polynucleotide Kinase (New England Biolabs, Ipswich, MA). Sense and antisense ONDs of each pair were mixed and annealed by heating at 98°C for 1 min and cooling off at room temperature for 1 h in a block incubator. Double-stranded ONDs, designated SBS1WT and SBS1MUT respectively, were purified with illustra ProbeQuant™ G-50 Micro Colum's (GE Healthcare, Little Chalfont, United Kingdom). Nuclear extracts were prepared from cells by using NE-PER Nuclear and Cytoplasmic Extraction Kit (Thermo Fisher Scientific Inc.). The radio-labeled DNA probe was incubated for 15 minutes at room temperature with the reaction mixtures, containing nuclear extract of U2OS (12 µg), 2 µl of 10× binding buffer (Thermo Fisher Scientific Inc.), 1 µg of poly(dI-dC), 2.5% glycerol, 5 mmol/L MgCl_2_, 1 mmol/L dithiothreitol and 0.5 mmol/L ZnCl_2_. DNA-protein complexes were loaded on a 6% nondenaturing polyacrylamide gel and electrophoresed at 200 V for 70 min. In the supershift assay, nuclear extracts were mixed with the anti-Sp1 antibody (1C6), the anti-Sp3 antibody (D-20), or rabbit non-immunized control IgG (Dako, Tokyo, Japan) in the reaction mixture and incubated for 30 min on ice before the formation of DNA-protein complexes. In the competition experiment, excess amounts of unlabeled ONDs were added to the reaction mixtures before the incubation with the labeled DNA probe.

### Chromatin Immunoprecipitation (ChIP) assay

Cells in a semi-confluent state in a 150-mm dish were fixed with formaldehyde at a final concentration of 1.0% for 10 min at room temperature to cross-link protein to DNA. Cells were washed with ice-cold PBS and lysed in 300 µl of lysis buffer (10 mM Tris-HCl pH 8.0, 300 mM NaCl, 1 mM EDTA, 0.5 mM EGTA, 0.1% sodium deoxycholate and 0.5% N-laurolysarcosine), then sonicated on ice. Triton-X 100 was added at a final concentration of 10% to dissolve the protein-DNA complexes. A soluble fraction was obtained after centrifugation at 20,000× g for 10 min at 4°C. Fifteen microliters of supernatant (one-twentieth of the total volume) was saved as an input, and the rest was divided into three and mixed with Dynabeads (Invtirogen) at 4°C overnight with rotation, which were pre-incubated with 2 µg of anti-Sp1 (PEP2) or -Sp3 (D-20) antibodies, or rabbit non-immune IgG at 4°C overnight. The next day, immunoprecipitated complexes were washed with low salt, high salt, LiCl RIPA buffer and finally with TE (pH 8.0) buffer containing 50 mM NaCl. The complexes were eluted from Dynabeads by treatment with the elution buffer (50 mM Tris-HCl pH 8.0, 10 mM EDTA and 1% SDS) and boiling at 65°C for 15 min. After centrifugation at 16,000× g for 15 min at room temperature, DNA-protein cross-links were reversed by incubating overnight at 65°C. Following RNaseA and proteinase K treatment, DNA was purified and precipitated with the phenol-chloroform method. PCR was performed to amplify a DNA fragment spanning from -136 to +142 including two SBSs and EBSs by KOD Plus polymerase (TOYOBO) and a set of primers listed in Table S1.

### Western blot analyses

Western blotting was performed as described previously [1]. Membranes were probed with anti-AFAP1L1 (1∶2000), anti-Sp1 (1C6, 1∶1000), anti-Sp3 (1∶1000), anti-β-tubulin (1∶1000), anti-acetyl H3K9 (1∶2500) and anti-Flag (1∶2000) antibodies. An NE-PER Kit (Thermo Fisher Scientific Inc.) was used to prepare nuclear protein before the Western blotting.

### Small inhibitory RNAs (siRNAs)

siRNA duplexes were transfected into cells (1.5×10^6^ cells) using RNAiMAX (Invitrogen) at a concentration of 20 nM. RNA and protein were extracted 48 h and 72 h after transfection, respectively. To knock down the *Sp1* and *Sp3* genes, two different siRNAs were used (siSp1#1 and siSp1#2 for Sp1; siSp3#1 and siSp3#2 for Sp3). siSp1#1 and siSp3#1were purchased from Dharmacon (Thermo Fisher Scientific Inc.) and had been used in our previous study [6]. Luciferase GL2 siRNA (siGL2) and GL3 siRNA (siGL3) were also purchased from Dharmacon. siSp1#2, siSp3#2, and an siRNA sequence targeting Sp4 gene (siSp4) were synthesized by Sigma-Aldrich (Table S1).

### siRNA-resistant Sp3 gene

A vector that harbors the *Sp3(li-1)* gene resistant to both siSp3#1 and siSp3#2 was generated by a mutagenesis-based method. Primers for mutagenesis were designed to harbor silent mutations at the third nucleotide of every codon in the target sequence (Table S1). pcDNA/Sp3(li-1) was sequentially mutated using the two sets of primers, and the construct was transferred to pLenti6/V5-DEST (Invitrogen). pLenti6/V5-GW/*lacZ* (Invitrogen) and pLenti6/V5-DEST/EGFP were used as lentiviral controls. Using the ViraPower Lentiviral Expression System (Invitrogen), U2OS cells were infected with viral supernatant containing the siRNA-resistant *Sp3(li-1)* or control gene according to the manufacturer's instructions.

### Matrigel invasion assay

At 48 h after siRNA treatment, cells were collected and cultured in BioCoat Matrigel Invasion Chambers (BD Biosciences) and 8-µm pore Control Cell Culture Inserts (BD Biosciences) as described previously [1]. Cells (5×10^4^) were seeded in each chamber in triplicate and incubated for 22 h. Then cells were fixed and migrating cells were counted in five random fields under the microscope at ×100 magnification.

## Results

### *AFAP1L1* mRNA expression in sarcoma cell lines

**Figure 1:**
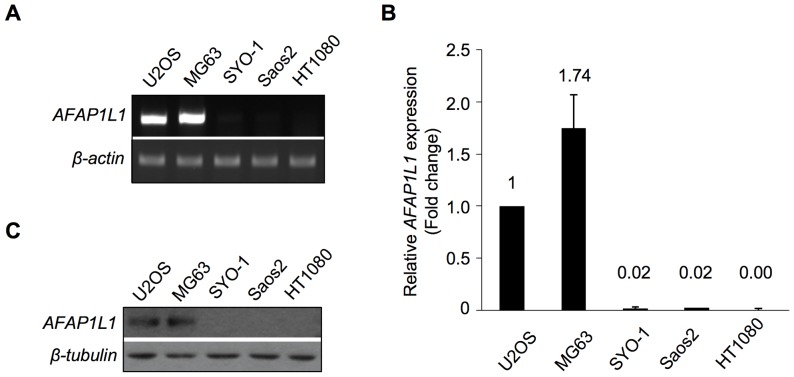
AFAP1L1 expression in sarcoma cell lines. (**A**) **mRNA expression of the** ***AFAP1L1*** **gene in sarcoma cell lines.** Reverse transcribed cDNA from each cell line was used as a template for PCR with primers specific for the *AFAP1L1* gene. The *β-actin* gene was used as a control. (B) Quantitative analysis of the gene expression of *AFAP1L1*. qPCR was performed with a Taqman probe and the primers listed in Table S1. Expression levels were calculated as fold changes relative to U2OS. (C) Protein expression of AFAP1L1. Total cell lysate from each cell line was used for Western blotting. β-tubulin was used as a control. Error bars indicate standard deviations.

First, we checked *AFAP1L1* expression in sarcoma cell lines by RT-PCR and qPCR. *AFAP1L1* was expressed strongly in U2OS and MG63 cells, very weakly in SYO-1 and Saos2 cells, and not at all in HT1080 cells (Fig. 1A–B). In the Western blot analysis, AFAP1L1 was detected in U2OS and MG63 cells but undetectable in SYO-1, Saos2 and HT1080 cells (Fig. 1C), indicating that the expression of AFAP1L1 was regulated differently among sarcomas at the transcriptional level.

### *AFAP1L1* promoter activity depends on the proximal conserved region

**Figure 2:**
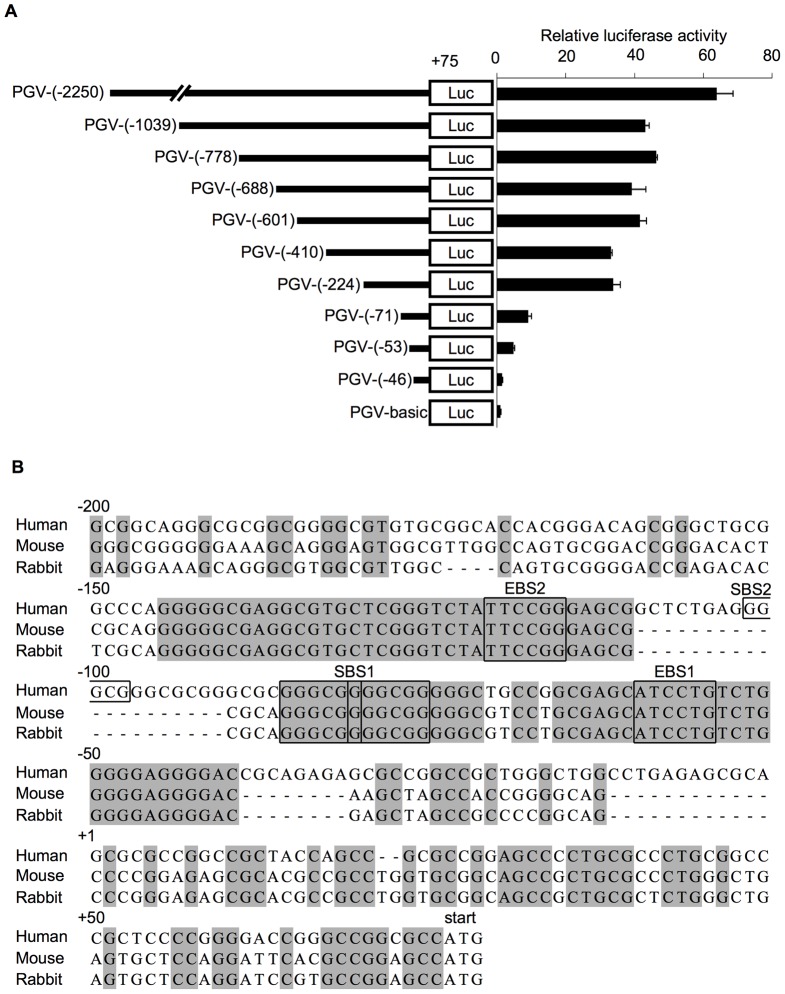
Identification of the core promoter region of the *AFAP1L1* gene. (A) Transcriptional activity of the 5′-flanking region of the *AFAP1L1* gene. Luciferase reporter assays were performed using a series of constructs carrying DNA fragments derived from the 5′-flanking region of the *AFAP1L1* gene. Numbers indicate the position relative to the transcriptional start site (TSS), and in all cases, the 3′ end of fragments was at the start codon, which was located 75 bases upstream of TSS. (B) Comparison of 5′-flanking region of the *AFAP1L1* gene among species. Human, mouse and rabbit sequences of the 5′-flanking region of the *AFAP1L1* gene are aligned, and conserved sequences are shown in gray boxes. EBS, Ets-binding site; SBS, Sp1-binding site.

To identify the transcriptional regulatory elements of the *AFAPL1* gene, DNA fragments with various segments of the *AFAP1L1* promoter were cloned into the PGV-basic vector as described in the section of Materials and Methods. They were transfected into U2OS cells expressing endogenous AFAP1L1 and their luciferase activities were measured (Fig. 2A). The longest fragment showed the strongest promoter activity and shorter ones less, but the decrease was not remarkable until the fragment lost the region between −224 and −71 relative to TSS (Fig. 2A). By searching the CONSITE database [7], we found that the sequence from −150 to −40 was highly conserved in three species (Fig. 2B). Of note, within that conserved region two Ets-binding motifs (5′-(A/C)GGA(A/T)-3′) and two Sp1-binding motifs (5′-GGGCGG-3′) were identified. The proximal (−60 to −56) and distal (−102 to −97) Ets-binding motifs were designated Ets-binding site 1 (EBS1) and 2 (EBS2), respectively. The proximal Sp1-binding site (−86 to −76) contained two overlapping consensus sequences (−86 to −81 and −81 to −76) and was conserved completely in all three species, and was designated SBS1. The distal Sp1-binding site (SBS2) spanning −102 to −97 was found only in the human genome. Several studies have shown that Ets and Sp proteins function together in the transcription of target genes [8], [9], and therefore we focused on Ets and Sp transcription factors.

### The Proximal Sp1-binding site is essential to *AFAP1L1* transcription

**Figure 3:**
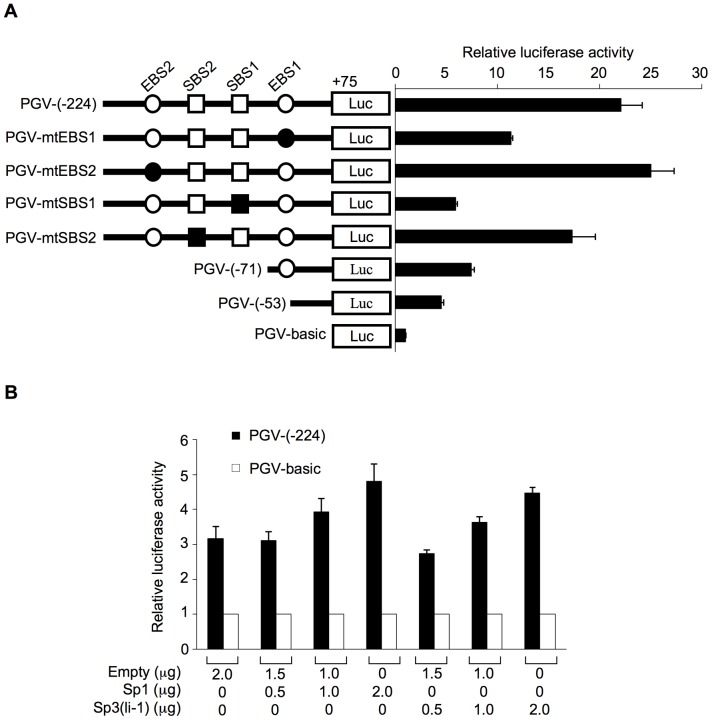
Identification of Sp1-binding sites as essential sequences for *AFAP1L1* transcription. (A) Identification of core domains for transcriptional activity. Open and closed circles represent wild-type and mutated EBS and open and closed rectangles represent wild-type and mutated SBS. PGV-vectors containing various segments of the *AFAP1L1* promoter were transfected into U2OS cells, and their luciferase activities were measured. (B) The effect of exogenous Sp1 and Sp3 on the transcriptional activity of the core promoter region of the *AFAP1L1* gene. The luciferase activity of the core promoter region (−224 to +75) was evaluated after Sp1 or Sp3-expressing vectors were co-transfected into U2OS cells. The total amount of transfected plasmid DNA was equalized by the addition of pcDNA3.1(+), an empty vector. Error bars indicate standard deviations.

To investigate the role of Ets and Sp transcription factors in the promoter activity, four types of luciferase reporters with mutations in the conserved sequence of each binding site were constructed using PGV-(−224) as a template and designated PGV-mtEBS1, PGV-mtEBS2, PGV-mtSBS1, and PGV-mtSBS2. When EBS1 was mutated, the promoter activity was reduced by 50% compared to PGV-(−224), although PGV-(−71) which retained EBS1 also showed reduced activity (Fig. 3A). However, the effect was most remarkable when SBS1 was mutated, which resulted in a 75% reduction in promoter activity (Fig. 3A). This level was almost equivalent to that of PGV-(−53), which retained no EBSs or SBSs. Mutations in EBS2 or SBS2 had less significant effects on the promoter activity (Fig. 3A). These results suggested that although both Sp and Ets proteins might play roles in transcriptional regulation of the *AFAP1L1* gene, the Sp protein binding to SBS1 is the main factor driving the expression of *AFAP1L1*. Therefore, we focused on Sp proteins.

### Sp1 and Sp3 transactivate the proximal *AFAP1L1* promoter

To determine whether Sp1 and/or Sp3 transactivate the promoter activity of the *AFAP1L1* gene, a luciferase assay was carried out using the Sp1 (pEVR2/Sp1) and Sp3 (pcDNA/Sp3(li-1)) expression vectors, which produce each protein effectively in transfected cells (Fig. S1). Co-transfection of the Sp1 or Sp3(li-1) expression vector increased the promoter activity of PGV-(−224) in a dose-dependent manner (Fig. 3B), suggesting Sp1 and Sp3 to function in the transactivation of *AFAP1L1*. Interestingly, co-transfection of the vector expressing a short form of Sp3, Sp3(si-1), significantly reduced the promoter activity of PGV-(−224) (Fig. S2). No significant effects were observed on the co-transfection of the Sp3(li-2) or Sp3(si-2) expression vector (data not shown).

### Sp1 and Sp3 bind to *AFAP1L1*'s proximal promoter region

**Figure 4:**
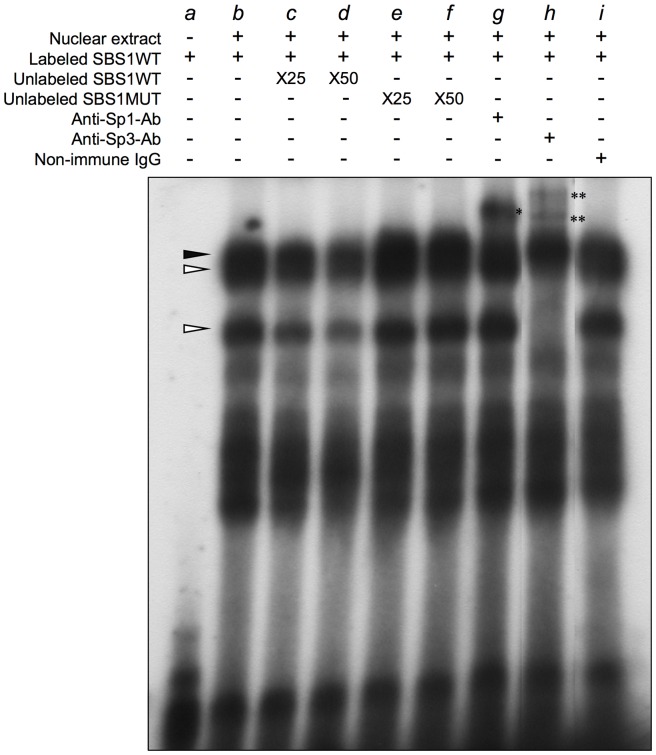
Binding of Sp transcription factors to the core-promoter region of the *AFAP1L1* gene *in vitro*. EMSA was performed to analyze the binding ability of putative transcription binding sites. Nuclear extracts were prepared from U2OS cells. Cold competitor experiments were conducted by the addition of 25- and 50-fold excess amounts of unlabeled SBS1WT or SBS1MUT to nuclear extracts before incubating with labeled SBS1WT (*lanes c*–*f*). Supershift experiments were conducted by the addition of anti-Sp1 or anti-Sp3 antibody to protein-OND complexes (lanes *g* and *h*). Non-immune IgG was used as a control (lane *i*). Open and closed arrowheads indicate the Sp3-OND and Sp1-OND complex, respectively. Single and double asterisks indicate bands supershifted by the addition of Sp1 or Sp3 antibody, respectively.

To elucidate whether Sp1 and Sp3 bind to SBS1 *in vitro*, EMSA was conducted using labeled SBS1 OND and U2OS nuclear extract. Using wild-type ONDs (SBS1WT), several shifted bands were observed (Fig. 4, *lane b*), among which three showed a decrease in intensity in competition with unlabeled SBS1WT in a dose-dependent manner (Fig. 4, *lanes c–d*). These three bands were not detected when labeled SBS1MUT was used instead of SBSWT for the assay (Fig. S3, *lanes f–h*). When unlabeled SBS1MUT was used as a competitor, no reduction in intensity was observed (Fig. 4, *lanes e* and *f*), suggesting that the bands were specific to SBS1 complexes. When the anti-Sp1 antibody was added to the OND/protein mixture, the intensity of the uppermost band decreased and a supershifted band was identified, whereas no remarkable changes were observed in the other two bands (Fig. 4, *lane g*; Fig. S3, *lane c*). The intensity of the uppermost band showed no change when an anti-Sp3 antibody was used but the other two bands showed a clear difference (Fig. 4, *lane h*; Fig. S3, *lane d*). The intensity of the middle band decreased and the lower band almost disappeared, which was associated with the appearance of two supershifted bands (Fig. 4, *lane h*). These changes were not observed when labeled SBS1MUT was used in the assay (Fig. S3, *lanes g*–*h*). No remarkable change was observed with the addition of control IgG (Fig. 4, *lane i*). These results suggested that the uppermost and lower two bands corresponded to Sp1- and Sp3-OND complexes, respectively, and therefore both Sp1 and Sp3 are able to bind to the proximal Sp1-binding site *in vitro*. Similar results were obtained when nuclear extracts were prepared from MG63 cells, which were strongly positive for AFAP1L1 (Fig. S4, *lanes h–n*). Interestingly, similar results were also obtained when nuclear extracts were prepared from SYO-1 cells, which were very weakly positive for AFAP1L1 (Fig. S4, *lanes a–g*). These results suggested that the expression of AFAP1L1 *in vivo* was regulated by not only the *cis*-element but also other factors such as chromatic modification.

### Sp3 regulates the transcription of the *AFAP1L1* gene by binding to the endogenous promoter region

**Figure 5:**
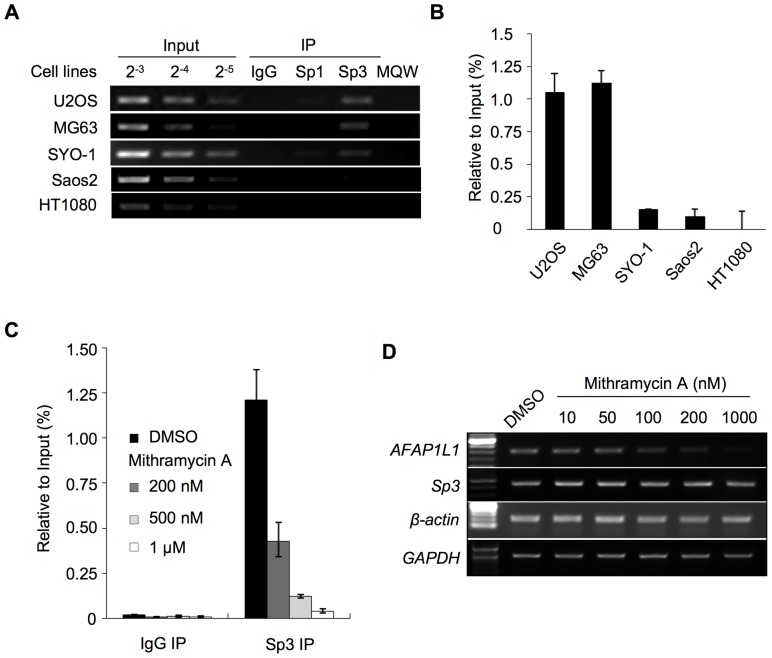
Identification of Sp3 as a major transcription factor for *AFAP1L1*. (A) and (B) Binding of Sp transcription factors to the core-promoter region of the *AFAP1L1* gene *in vitro*. ChIP assays were performed using anti-Sp1 and anti-Sp3 antibodies or control IgG and the precipitated DNA was PCR-amplified using a pair of primers located in the core-promoter region (Table S1) (A), and the precipitated genome was quantified by qPCR (B). (C) The effect of mithramycin A treatment on Sp3 binding. U2OS cells were treated with mithramycin A or DMSO for 48 h, and immunoprecipitated DNA by Sp3 antibody was quantified by qPCR. (D) The effect of mithramycin A on the expression of the *AFAP1L1* gene. RNA was extracted from U2OS cells treated with mithramycin A or DMSO for 48 h, and RT-PCR was performed to semi-quantify the expression of each gene. The *β-actin* and *GAPDH* genes were used as a control. Error bars indicate standard deviations.

To investigate whether Sp1 and/or Sp3 bind to SBS1 *in vivo*, ChIP assays were conducted using four cell lines in which the gene expression of AFAP1L1 differed considerably; U2OS (strong), MG63 (strong), SYO-1 (very weak), Saos2 (very weak) and HT1080 (null) (Fig. 1A). We found that Sp3 bound to the *AFAP1L1* promoter region strongly in U2OS and MG63 cells (Fig. 5A), but weakly in SYO-1 and Saos2 cells. No binding of Sp3 to the proximal promoter region was detected in HT1080 cells. Binding of Sp1 was below the significant level by as determined by qPCR (data not shown). Quantitative analyses showed a clear correlation between the binding of Sp3 and the expression level of *AFAP1L1* (Fig. 1A and Fig. 5B). To exclude the possibility that this difference in the binding of Sp3 to the promoter is due to mutations in binding sites, we checked the genomic DNA of U2OS, MG63, SYO-1 and HT1080. No mutations were found in the proximal promoter including EBS1, EBS2, SBS1 and SBS2 in any of the cell lines investigated (data not shown).

Mithramycin A is an aureolic acid antibiotic, which inhibits gene expression by displacing transcriptional activators like the Sp protein family that bind to GC-rich regions of promoters [10], [11]. Treatment with mithramycin A inhibited the binding of Sp3 to the promoter region of the *AFAP1L1* gene in a dose-dependent manner (Fig. 5C). Consistent with this finding, the treatment with Mithramycin A reduced the mRNA expression of *AFAP1L1* without changing that of Sp3 in U2OS cells (Fig. 5D). Similar results were observed in another AFAP1L1-positive cell line, MG63 cells (Fig. S5). These results indicate that the binding of Sp3 to SBS1 is a prerequisite for *AFAP1L1* transcription, the level of which is regulated by the extent of the binding. Total and nuclear protein levels of Sp3 are almost the same in these four cell lines (Fig. S6A–B), suggesting the existence of undiscovered mechanisms that regulate the binding of Sp3 to SBS1. The luciferase assays suggested the involvement of the Ets protein family in the regulation of *AFAP1L1* transcription (Fig. 3A). Transfection of a dominant-negative Ets vector significantly reduced *AFAP1L1* promoter activity, also suggesting the Ets family to participate in the transcription of *AFAP1L1* (Fig. S7A). Interestingly, transfection of ELK1, another member of the Ets family, reduced *AFAP1L1* promoter activity (Fig. S7A), and we found that forced expression of ELK1 up-regulated the two short isoforms of Sp3 (Fig. S7B), which may be responsible for the reduction in promotor activity, based on the results of co-transfection experiments (Fig. S2). mRNA expression levels of *ELK1* and *ELK4* showed no significant differences among sarcoma cell lines irrespective of the AFAP1L1 expression level (Fig. S7C).

### Sp3 is essential to the expression of AFAP1L1

**Figure 6:**
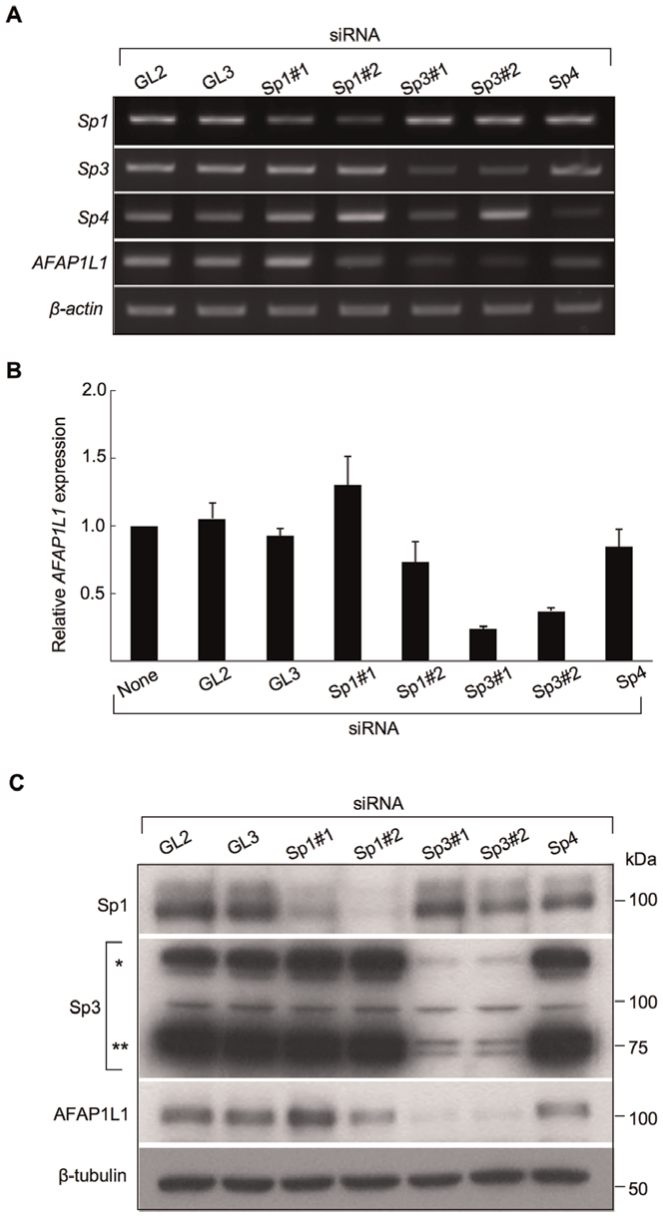
Linking of Sp3 with AFAP1L1 by siRNA experiments. (A) The specificity of siRNA. U2OS cells were treated with siRNA targeting *Sp1*, *Sp3*, or *Sp4* for 48 h, and the expression of these genes as well as the *AFAP1L1* gene was analyzed by PCR. Two different siRNAs targeting the *Sp1* and *Sp3* genes were designed and used. *β-actin* was used as a control. (B) Down-regulation of *AFAP1L1* expression by siRNA targeting the *Sp3* gene at the mRNA level. U2OS cells were treated with siRNAs targeting each gene for 48 h and the expression of *AFAP1L1* was analyzed by qPCR and indicated as fold changes relative to that in untreated cells. (C) Down-regulation of AFAP1L1 expression by siRNA targeting the *Sp3* gene at the protein level. U2OS cells were treated with siRNA targeting each gene for 72 h and proteins were extracted and used for Western blotting. β-tubulin was used as a control.

**Figure 7:**
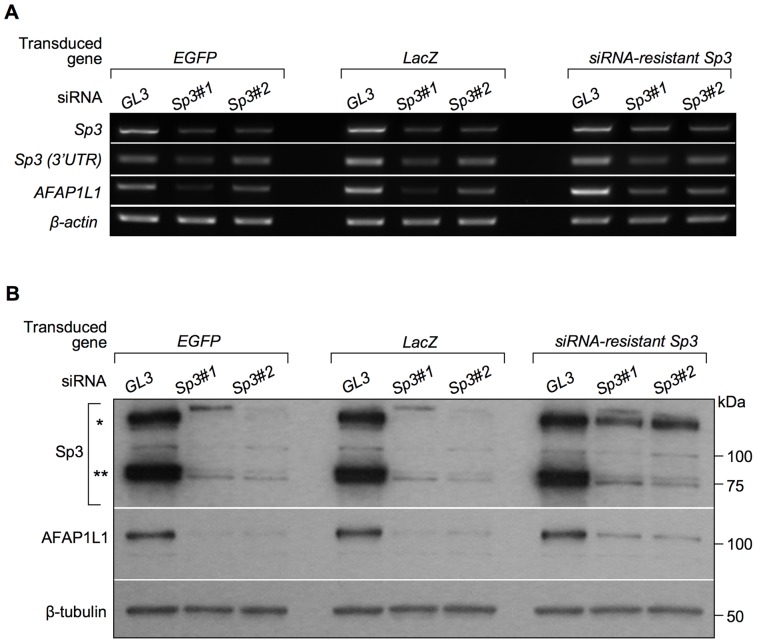
Restoration of down-regulated AFAP1L1 expression by an siRNA-resistant Sp3 expression vector. U2OS cells stably expressing the Sp3 mRNA resistant to Sp3#1 and Sp3#2 siRNA was established and treated with these siRNAs. U2OS cells stably expressing the *EGFP* or *LacZ* gene were employed as a control. After 48-h-treatment with siRNAs, RNA was extracted from each cell and the expression of *Sp3* and *AFAP1L1* was analyzed by RT-PCR (A). Knocking down of the endogenous *Sp3* gene was confirmed by PCR using a set of primers located in the 3′ UTR of the *Sp3* gene (Table S1). The *β-actin* gene was used as a control. Protein was extracted after 72 h of treatment and used for Western blotting (B). β-tubulin was used as a control. Error bars indicate standard deviations. Single and double asterisks indicate the long and short forms of the Sp3 protein, respectively.

Finally, siRNA was employed to investigate the role of Sp3 in AFAP1L1 transcription *in vivo*. In U2OS cells, siRNA targeting each of Sp1, Sp3, and Sp4 significantly reduced the expression of the targeted gene, but only the siRNA targeting Sp3 consistently reduced the expression of the *AFAP1L1* gene (Fig. 6A), which was confirmed by quantitative analyses (Fig. 6B). Specific reduction of *AFAP1L1* expression by siRNA against Sp3 was further confirmed at the protein level (Fig. 6C). These effects of siRNA against Sp3 were also confirmed in other cell lines (MG63 and SYO-1) at the mRNA level (Fig. S8A–D). This phenomenon was also observed in prostate cancer PC-3 cells (Fig. S9), indicating that the transcriptional role of Sp3 for the *AFAP1L1* gene is not restricted to sarcoma cells. To exclude the off-target effect of siRNA, a rescue experiment was carried out. Pre-induction of siRNA-resistant Sp3 using a lentivirus partially rescued *AFAP1L1* expression after Sp3 siRNA treatment (Fig. 7A–B), indicating that the reduction in AFAP1L1 expression cause by siRNA for Sp3 is due to a direct effect on the Sp3 gene.

### Functional relevance of Sp3 to AFAP1L1

**Figure 8:**
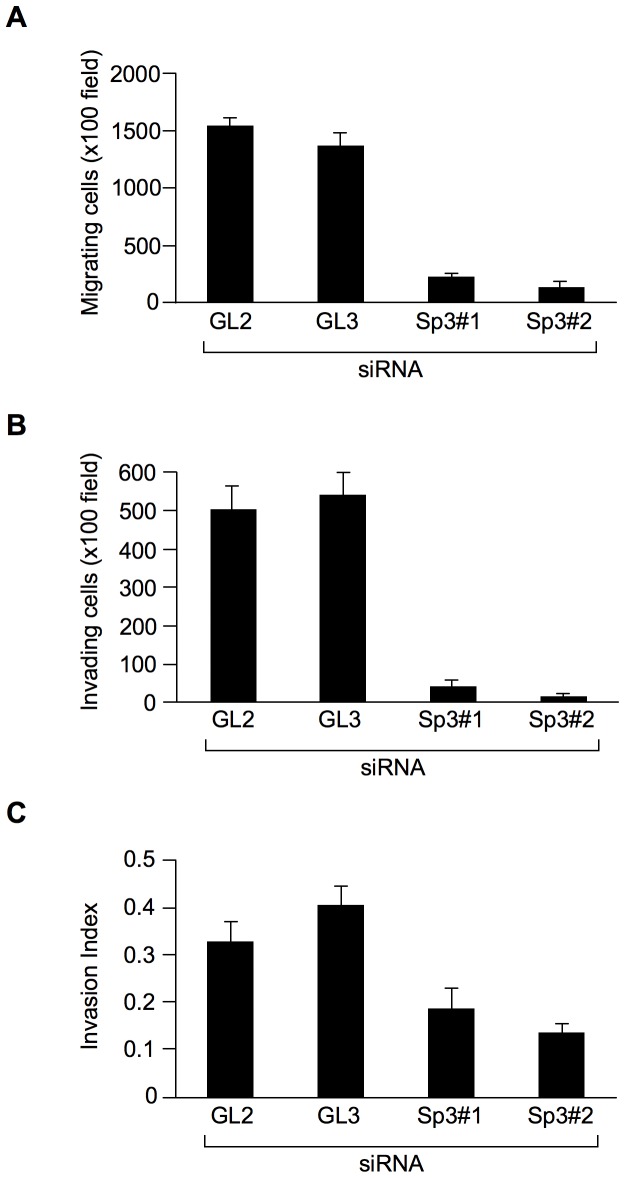
Inhibition of Sp3 expression reduces cell migration and invasiveness in U2OS cells. Numbers of cells migrating through the uncoated 8-micron membrane pores (A) and through the Matrigel-coated membranes (B) were counted in five randomly chosen fields at a magnification of ×100. (C) A cell invasion index was calculated as the ratio of the number of cells migrating through the matrigel to the number migrating through the uncoated membrane.

We have shown that the induction of AFAP1L1 expression increased cell motility and invasiveness in sarcoma cells [1]. Inhibition of Sp3 expression with siRNA also reduced the motility and invasiveness of U2OS cells, suggesting a functional link between Sp3 and AFAP1L1 (Fig. 8).

## Discussion

In the present study, we have found that Sp3 plays a critical role in the transcription of *AFAP1L1*, a gene associated with the metastasis of soft tissue spindle cell sarcomas [1]. Based on structural similarity, AFAP1, AFAP1L1 and AFAP1L2 belong to a family of new adaptor proteins. They all contain two pleckstrin homology domains flanking a serine/threonine-rich region, two Src homology (SH) 2-binding motifs and one or two SH3-binding motifs [1] [12] [13]. AFAP1, also known as AFAP-110, the most intensively investigated member of the family, is reported to have an intrinsic ability to alter actin filament integrity and may function as an adaptor protein by linking the Src family and/or other signaling proteins to actin filaments [13]. AFAP1L2, also termed XB130, has been cloned as an adaptor protein and Src kinase substrate and phosphorylated by RET/PTC, a genetically rearranged, constitutively active, thyroid- specific tyrosine kinase [14]. In contrast to AFAP1 and AFAP1L2, little is known about AFAP1L1. A recent study revealed that AFAP1L1 interacted with the SH3 domain of cortactin, an F-actin-binding protein [15] Although we had previously reported that AFAP1L1 was associated with the progression of sarcomas, how it functions in the invasiveness of tumor cells remains ill defined.

Sp3 is a member of the Sp/Kruppel-like factor (KLF) family. The Sp/KLF family recognizes GC/GT boxes and interacts with DNA through three zinc finger motifs [16]. Eight members of the Sp family, Sp1-8, have been reported. Sp1 was the first transcription factor identified and cloned among Sp family members [17] and has been intensively investigated. Since the DNA-binding domains of Sp1 and Sp3 share 90% homology in DNA sequence, they bind to the same DNA-binding site with similar affinity [16]. In spite of extensive studies on the Sp proteins, the difference in binding properties between Sp1 and Sp3 remains largely unknown. Notably, one study shows that promoters containing multiple adjacent Sp-binding sites form significantly more stable Sp3-DNA complexes than those with single Sp-binding sites, and as a consequence, Sp3 efficiently displaces Sp1 from preformed Sp1-DNA complexes from such sites [18]. Therefore, in *AFAP1L1*'s promoter region, the Sp3-SBS1 complexes might be more more stable than the Sp1-SBS1 complexes, because SBS1 contains two overlapping consensus Sp-binding sequences. The Sp3 protein has four isoforms; two long isoforms and two short isoforms [5]. All of them are derived from alternative translational start sites. The two long isoforms can act as transcriptional activators in certain settings, but the significance of the two small isoforms as transcriptional activators or inhibitors remains to be elucidated [5]. While investigating the role of Sp3 and Ets in the *AFAP1L1* promoter's activity, we found that forced expression of ELK1, an Ets transcription factor, induced up-regulation of the two short isoforms of Sp3 and resulted in decreased *AFAP1L1* promoter activity (Fig. S7B). As forced expression of a short isoform (si-1) reduced the *AFAP1L1* promoter activity induced by endogenous factors (Fig. S2), si-1 may have a negative effect on the transcription of *AFAP1L1*.

Sp1 and Sp3 have been shown to be expressed ubiquitously and reported to regulate basal and constitutive expression of genes both in normal and cancerous tissues [19]. Several reports have referred to a correlation between Sp1 and Sp3 and tumor development, growth and metastasis. Sp1 is reported to be overexpressed and regulate vascular endothelial growth factor (VEGF) in gastric and shown to be linked to a poor prognosis [20]. Up-regulation of Sp1 expression has been also observed in thyroid [21] and colorectal cancer [22]. Sp3 enhances the growth of pancreatic cancer cells by suppressing p27 expression through interaction with GC-rich promoter elements [23]. In breast cancer, Sp3 accelerates tumor cell growth by acting as a repressor of TGF signaling [24]. A recent report demonstrated the expression of Sp3 to be an independent prognostic factor for the poor survival of head and neck cancer patients [25]. Of note, in the web database ONCOMINE (http://www.oncomine.org), upregulation of Sp3 expression in soft tissue sarcomas compared to normal connective tissue has been confirmed [26] [27].

Because the cause of sarcoma patients' death is uncurable distant metastasis in most cases, methods of both predicting and treating metastasis are urgently needed. Our findings may provide new insight regarding this clinical difficulty. Considering that Sp3 is expressed at higher levels in soft tissue sarcomas and transactivates the *AFAP1L1* gene, targeting Sp3 could be a powerful approach to treating advanced soft tissue sarcomas.

## Supporting Information

Figure S1 **Expression of exogenous Sp1 or Sp3 protein in 293T cells.** 293T cells were transfected with each plasmid, as described in Materials and Methods, and the expression of the Sp1 or Sp3 protein was analyzed 24 h later. pRC/Sp3 lacks N-terminal part of the *Sp3* gene as described in *Experimental Procedures*. β-tubulin was used as an internal control. Single and double asterisks indicate the long and short forms of the Sp3 protein, respectively.
(TIF)

Figure S2 **Isoform-dependent activity of Sp3 on** ***AFAP1L1*** **promoter.** The luciferase reporter assay was performed as described in Fig. 3B. Reporter plasmids were co-transfected with either an empty, Sp1 or Sp3 expression vector. Error bars indicate the standard deviations.
(TIF)

Figure S3 **Binding of Sp transcription factors to the wild-type, but not mutated Sp-binding site** ***in vitro*** **.** Nuclear extracts were prepared from U2OS cells and used for EMSA with radiolabeled SBS1WT (*lane a–d*) or SBS1MUT (*lanes e–h*). A supershifted assay was performed with anti-Sp1 (*lane c* and *g*) or anti-Sp3 (*lane d* and *h*) antibody. Open and closed arrowheads indicate an Sp3-OND and Sp1-OND complex.
(TIF)

Figure S4 **EMSA using nuclear extracts from cells expressing the** ***AFAP1L1*** **gene very weakly (SYO-1) and strongly (MG63).** Nuclear extracts were prepared from SYO-1 and MG63 cells, and EMSA was performed as described in Figure 4. Open and closed arrowheads indicate Sp3-OND and Sp1-OND complex, respectively. Single and double asterisks indicate bands supershifted by the addition of Sp1 or Sp3 antibody, respectively.
(TIF)

Figure S5 **The effect of mithramycin in MG63 cells.** RNA was extracted from MG63 cells treated with mithramycin A at the indicated dose or DMSO for 48 h, and subjected to RT-PCR. The *β-actin* gene was used as a control.
(TIF)

Figure S6 **Western blot analyses of AFAP1L1, Sp1 and Sp3 in sarcoma cell lines.** Total cell lysate (A) or nuclear extract (B) was prepared from each cell line and used for Western blotting. β-tubulin and acetylated H3K9 were used as the internal control for total cell lysate and nuclear extract, respectively. Single and double asterisks indicate the long and short forms of the Sp3 protein, respectively.
(TIF)

Figure S7 **The effect of Ets transcription factors on the expression of** ***AFAP1L1*** **.** (A) The effect of Ets transcription factors on luciferase activity. Luciferase assays were performed in U2OS cells 48 h after the co-transfection of various expression vectors containing an Ets transcription factor with PGV-(−224). (B) The effect of ELK1 on the expression of Sp3. 293T cells were transfected with indicated plasmids and proteins were analyzed at 24 h by Western blotting. β-tubulin was used as an internal control. Single and double asterisks indicate the long and short forms of Sp3, respectively. DN-Ets represents dominant negative Ets. (C) Expression of ELK family gene in sarcoma cells. RNA was extracted from cells and RT-PCR was performed.
(TIF)

Figure S8 **Down-regulation of** ***AFAP1L1*** **expression by siRNA targeting the** ***Sp3*** **gene in SYO-1 and MG63 cells.** (A) and (D) The specificity of siRNA. SYO-1 (A) and MG63 (D) cells were treated with siRNA targeting *Sp1*, *Sp3*, or *Sp4* for 48 h, and the expression of these genes as well as the *AFAP1L1* gene was analyzed by PCR. Two different siRNAs targeting the *Sp1* and *Sp3* genes were designed and used. *β-actin* was used as a control. (B) and (E) Down-regulation of *AFAP1L1* expression by siRNA targeting the *Sp3* gene at the mRNA level. SYO-1 (B) and MG63 (E) cells were treated with siRNAs targeting each gene for 48 h and the expression of *AFAP1L1* was analyzed by qPCR and indicated as fold changes relative to that in untreated cells. (C) and (F) Down-regulation of AFAP1L1 expression by siRNA targeting the *Sp3* gene at the protein level. SYO-1 (C) and MG63 (F) cells were treated with siRNAs targeting each gene for 72 h and proteins were extracted and used for Western blotting. β-actin was used as a control.
(TIF)

Figure S9 **Down-regulation of Sp3 expression causes down-regulation of AFAP1L1 expression in prostate cancer cells.** (A) The specificity of siRNA. PC-3 cells were treated with siRNA targeting *Sp1*, *Sp3*, or *Sp4* for 48 h, and the expression of these genes as well as the *AFAP1L1* gene was analyzed by PCR. Two different siRNAs targeting the *Sp1* or *Sp3* gene were designed and used. *β-actin* was used as a control. (B) Down-regulation of AFAP1L1 expression by siRNA targeting the *Sp3* gene at the protein level. PC-3 cells were treated with siRNA targeting each gene for 72 h and proteins were extracted and used for Western blotting. β-tubulin was used as a control. Single and double asterisks indicate the long and short forms of Sp3, respectively.
(TIF)

Table S1 **Sequences for primers and other oligonucleotides used in this study.**
(XLS)
