# Supplementary figures and images for: The Transcription Factor Sp3 Regulates the Expression of a Metastasis-Related Marker of Sarcoma, Actin Filament-Associated Protein 1-Like 1 (AFAP1L1)
Source: PLoS One. 2013 Jan 9;8(1):e49709. doi: 10.1371/journal.pone.0049709 (PMC3541374; doi:10.1371/journal.pone.0049709)

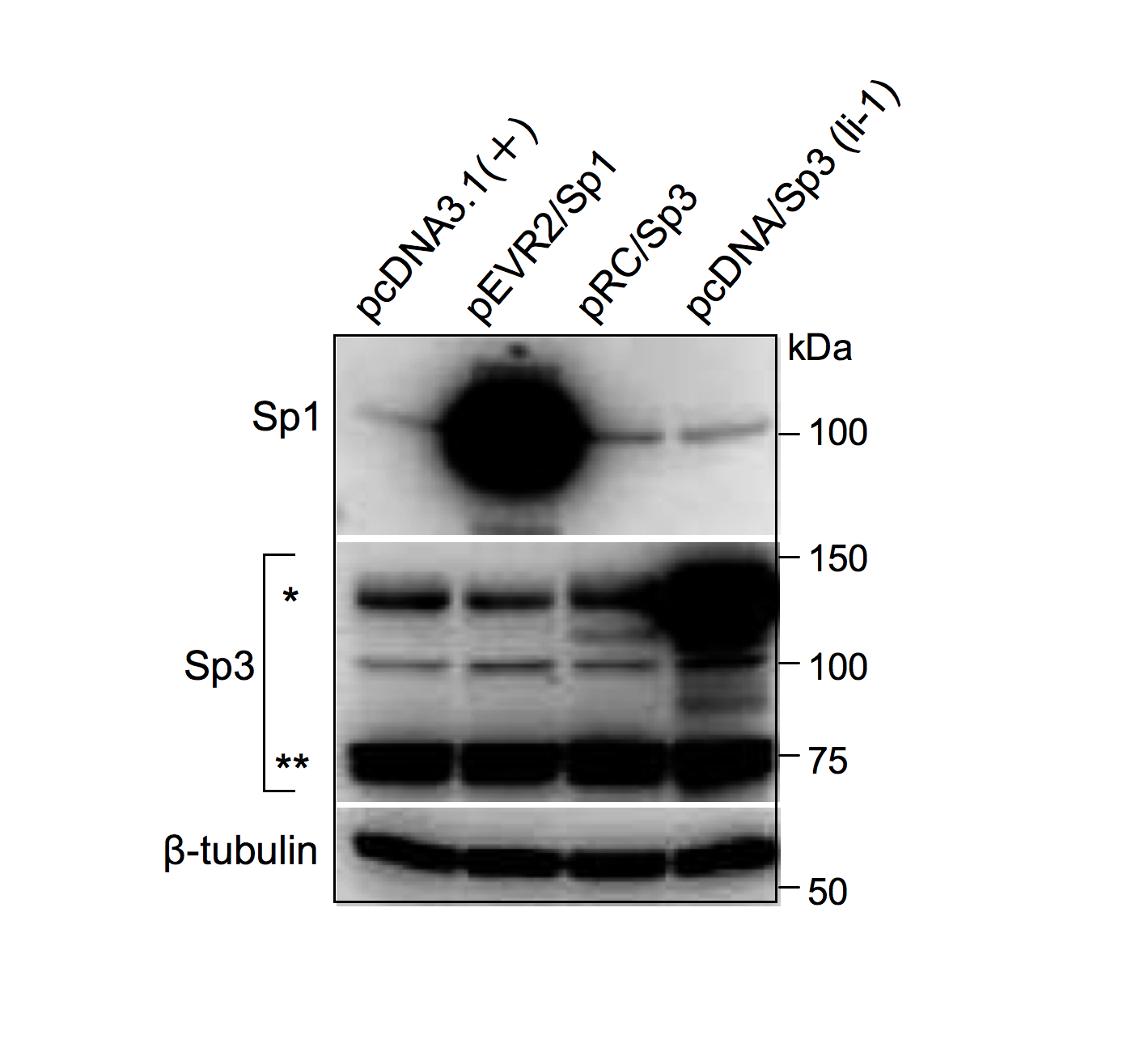

Supplement: Figure S1 — Expression of exogenous Sp1 or Sp3 protein in 293T cells. 293T cells were transfected with each plasmid, as described in Materials and Methods, and the expression of the Sp1 or Sp3 protein was analyzed 24 h later. pRC/Sp3 lacks N-terminal part of the Sp3 gene as described in Experimental Procedures. β-tubulin was used as an internal control. Single and double asterisks indicate the long and short forms of the Sp3 protein, respectively. (TIF) [file pone.0049709.s001.tif]

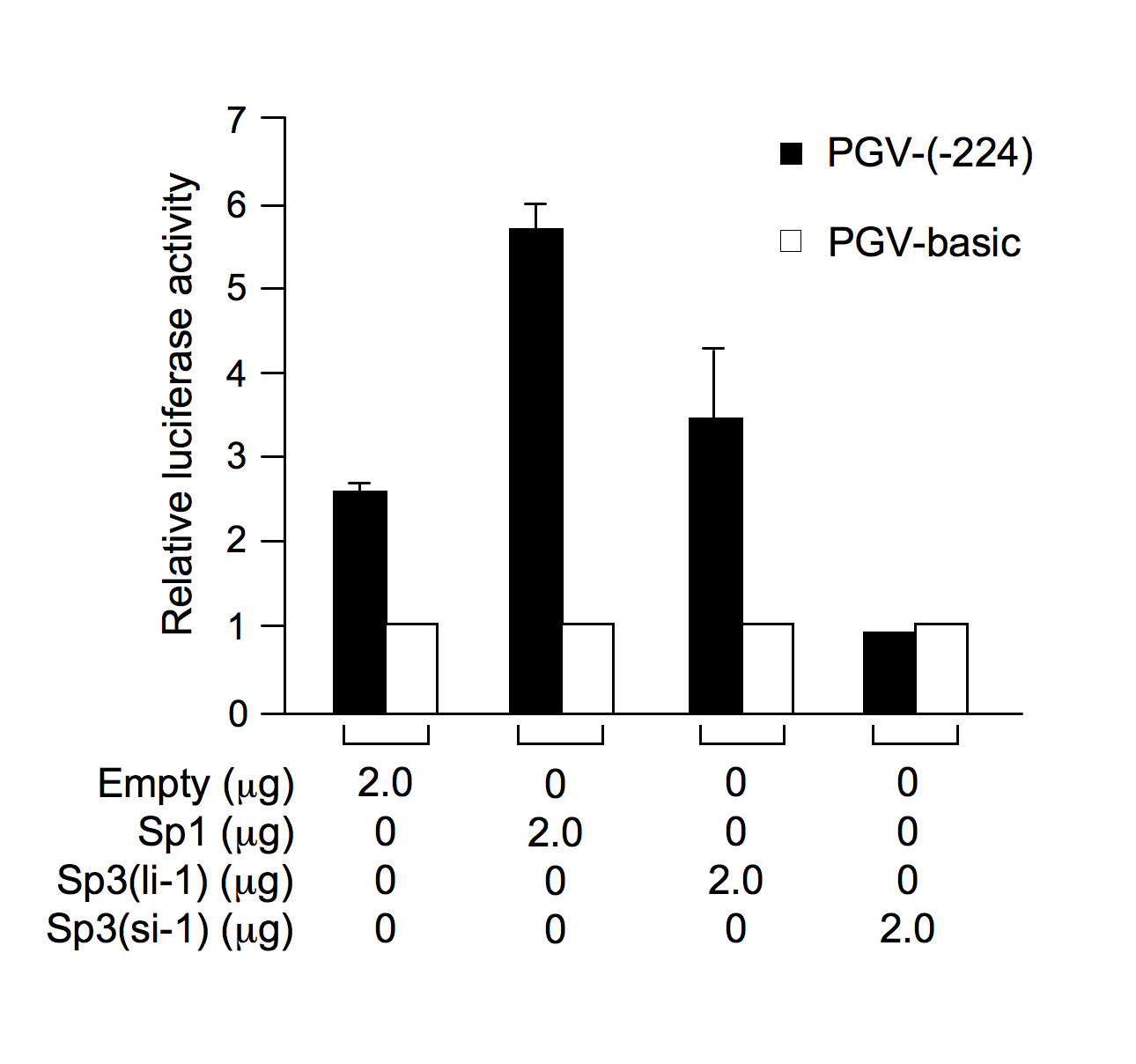

Supplement: Figure S2 — Isoform-dependent activity of Sp3 on AFAP1L1 promoter. The luciferase reporter assay was performed as described in Fig. 3B. Reporter plasmids were co-transfected with either an empty, Sp1 or Sp3 expression vector. Error bars indicate the standard deviations. (TIF) [file pone.0049709.s002.tif]

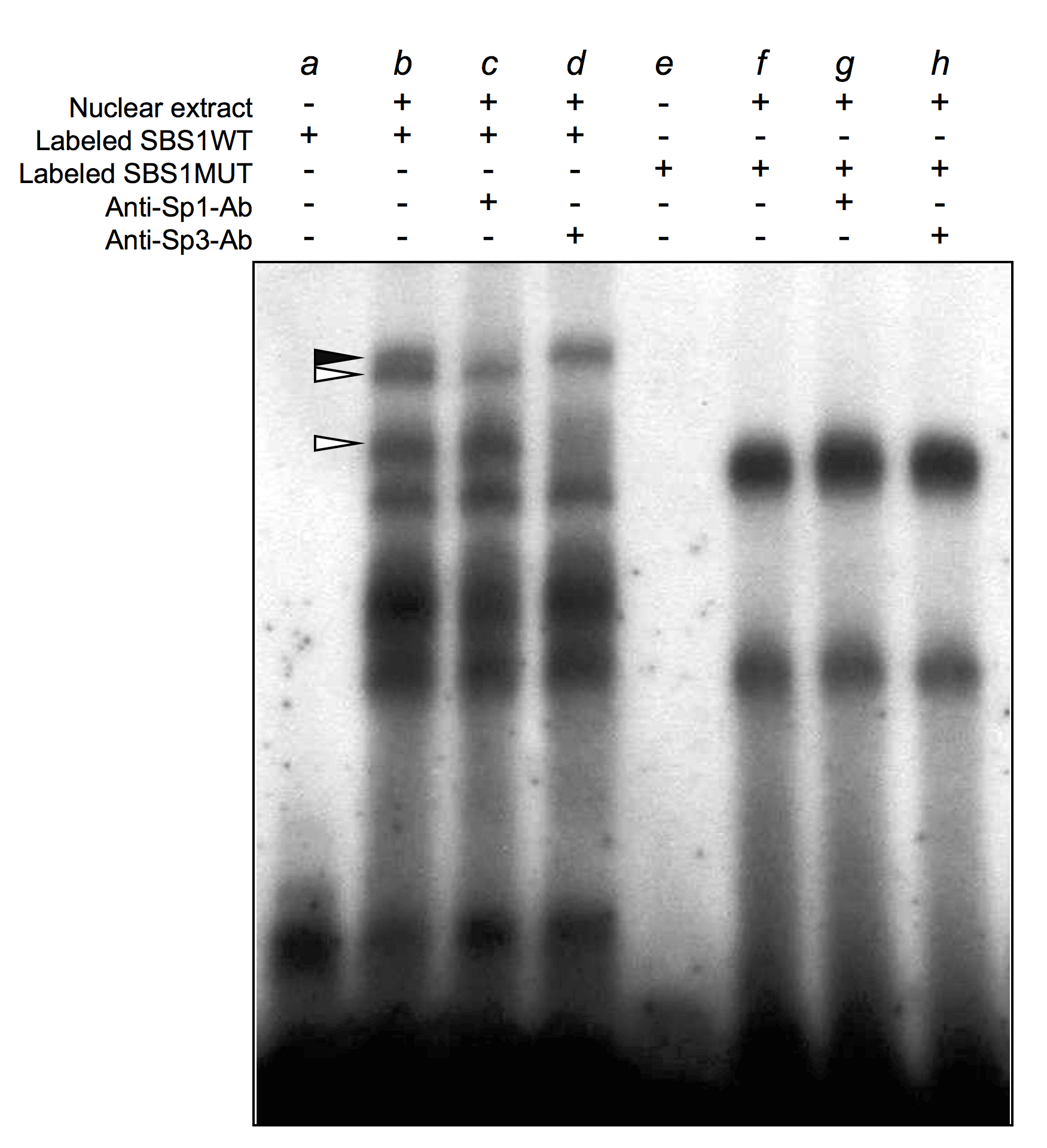

Supplement: Figure S3 — Binding of Sp transcription factors to the wild-type, but not mutated Sp-binding site in vitro . Nuclear extracts were prepared from U2OS cells and used for EMSA with radiolabeled SBS1WT (lane a–d) or SBS1MUT (lanes e–h). A supershifted assay was performed with anti-Sp1 (lane c and g) or anti-Sp3 (lane d and h) antibody. Open and closed arrowheads indicate an Sp3-OND and Sp1-OND complex. (TIF) [file pone.0049709.s003.tif]

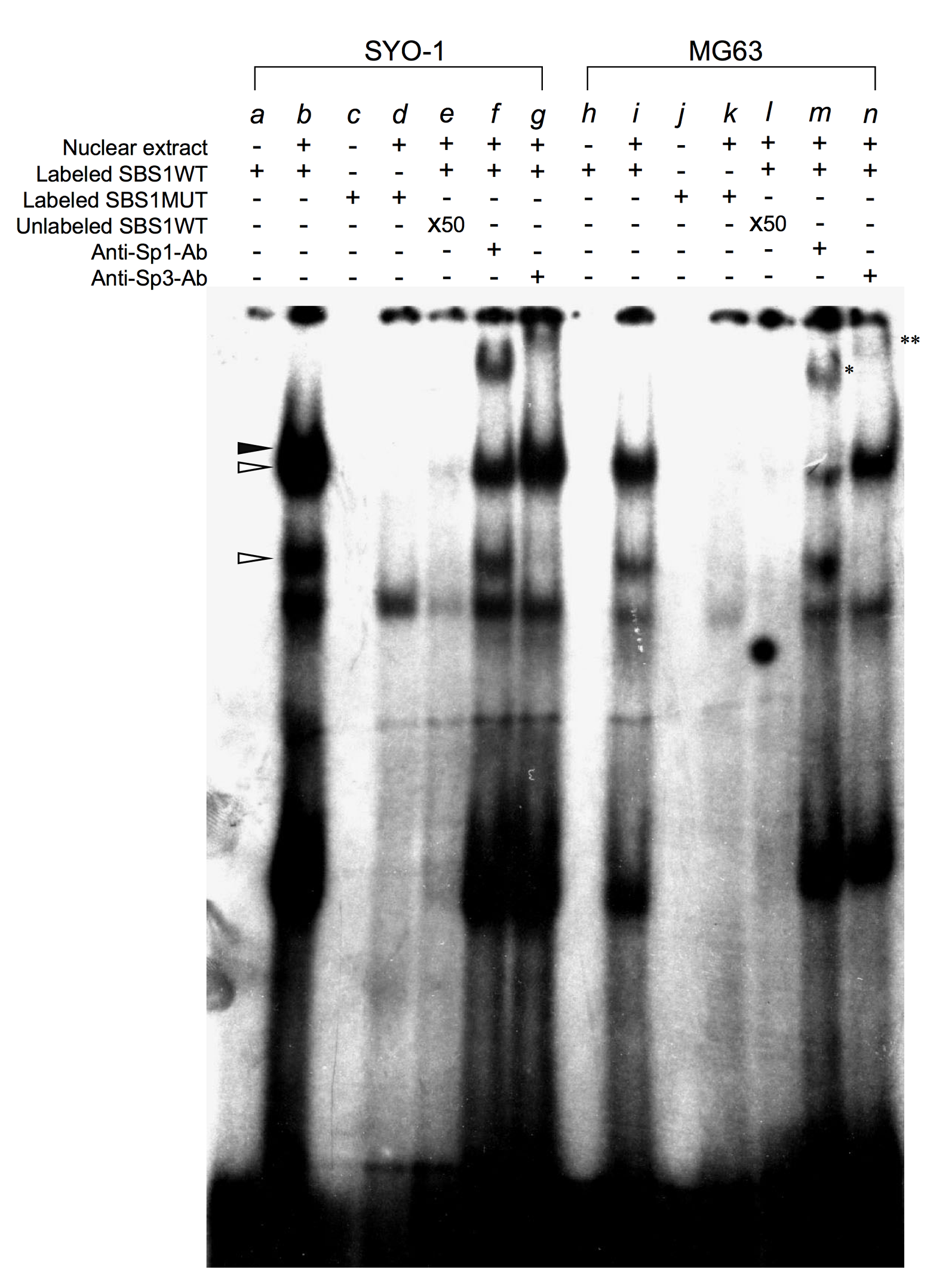

Supplement: Figure S4 — EMSA using nuclear extracts from cells expressing the AFAP1L1 gene very weakly (SYO-1) and strongly (MG63). Nuclear extracts were prepared from SYO-1 and MG63 cells, and EMSA was performed as described in Figure 4. Open and closed arrowheads indicate Sp3-OND and Sp1-OND complex, respectively. Single and double asterisks indicate bands supershifted by the addition of Sp1 or Sp3 antibody, respectively. (TIF) [file pone.0049709.s004.tif]

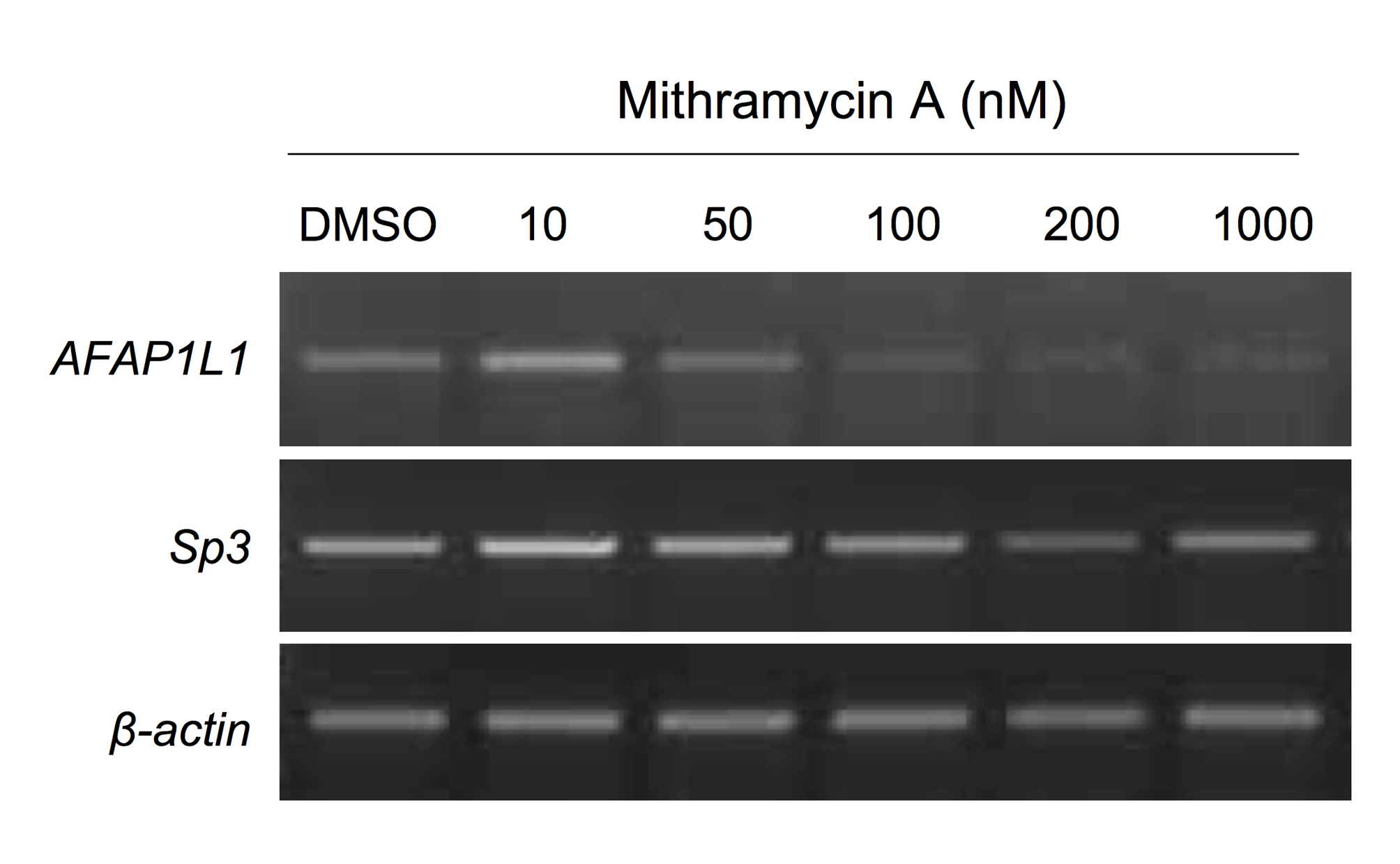

Supplement: Figure S5 — The effect of mithramycin in MG63 cells. RNA was extracted from MG63 cells treated with mithramycin A at the indicated dose or DMSO for 48 h, and subjected to RT-PCR. The β-actin gene was used as a control. (TIF) [file pone.0049709.s005.tif]

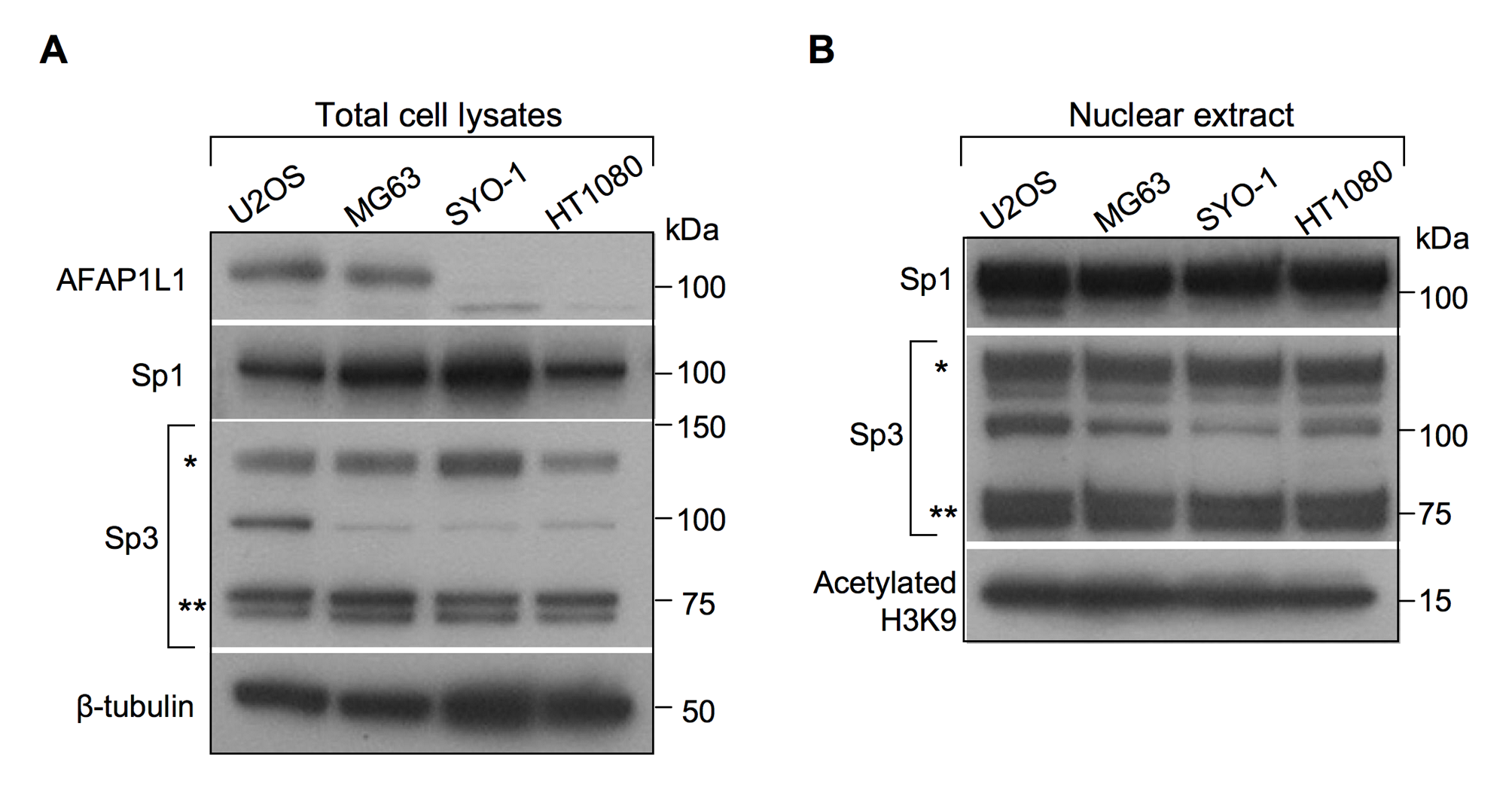

Supplement: Figure S6 — Western blot analyses of AFAP1L1, Sp1 and Sp3 in sarcoma cell lines. Total cell lysate (A) or nuclear extract (B) was prepared from each cell line and used for Western blotting. β-tubulin and acetylated H3K9 were used as the internal control for total cell lysate and nuclear extract, respectively. Single and double asterisks indicate the long and short forms of the Sp3 protein, respectively. (TIF) [file pone.0049709.s006.tif]

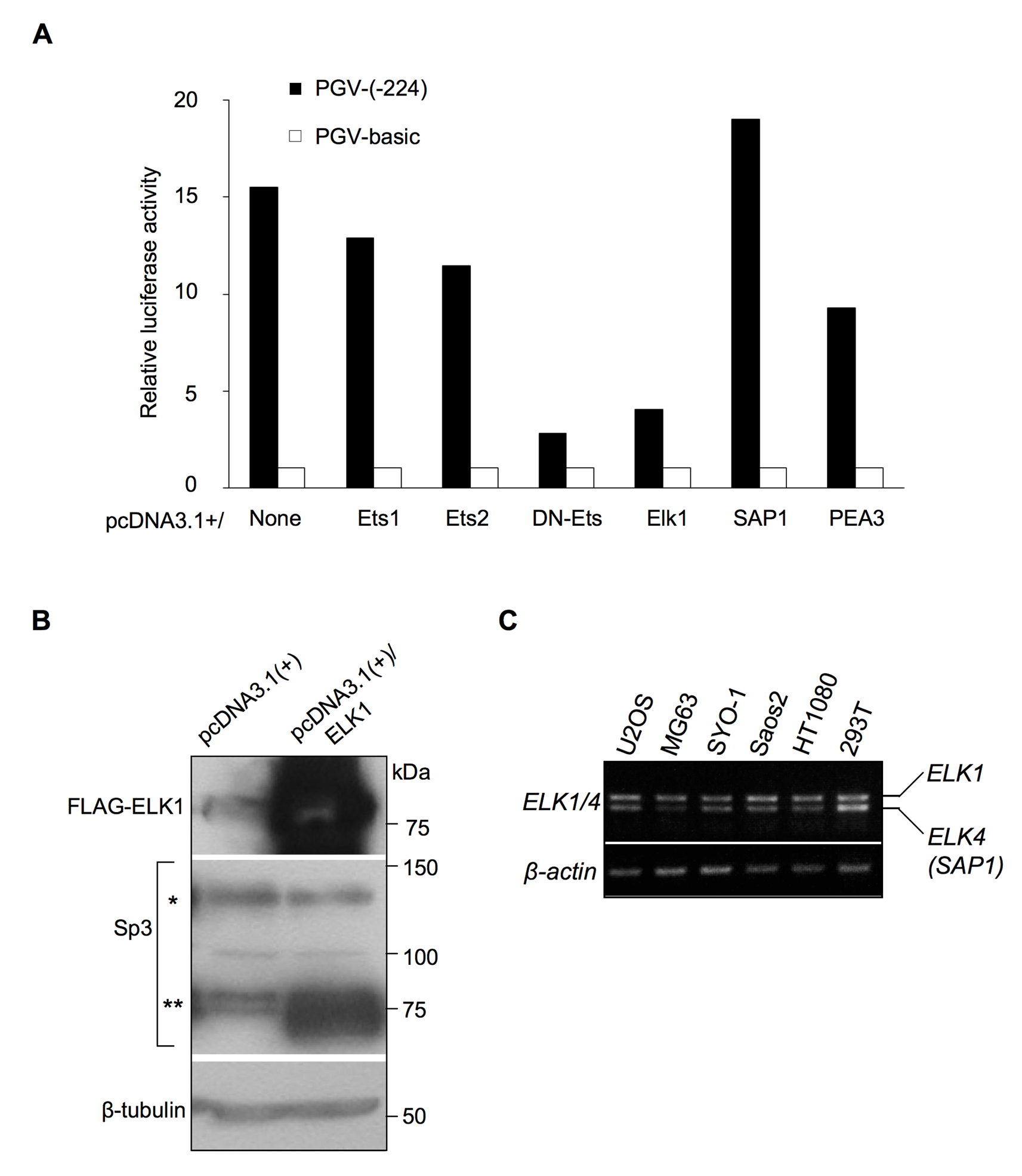

Supplement: Figure S7 — The effect of Ets transcription factors on the expression of AFAP1L1 . (A) The effect of Ets transcription factors on luciferase activity. Luciferase assays were performed in U2OS cells 48 h after the co-transfection of various expression vectors containing an Ets transcription factor with PGV-(−224). (B) The effect of ELK1 on the expression of Sp3. 293T cells were transfected with indicated plasmids and proteins were analyzed at 24 h by Western blotting. β-tubulin was used as an internal control. Single and double asterisks indicate the long and short forms of Sp3, respectively. DN-Ets represents dominant negative Ets. (C) Expression of ELK family gene in sarcoma cells. RNA was extracted from cells and RT-PCR was performed. (TIF) [file pone.0049709.s007.tif]

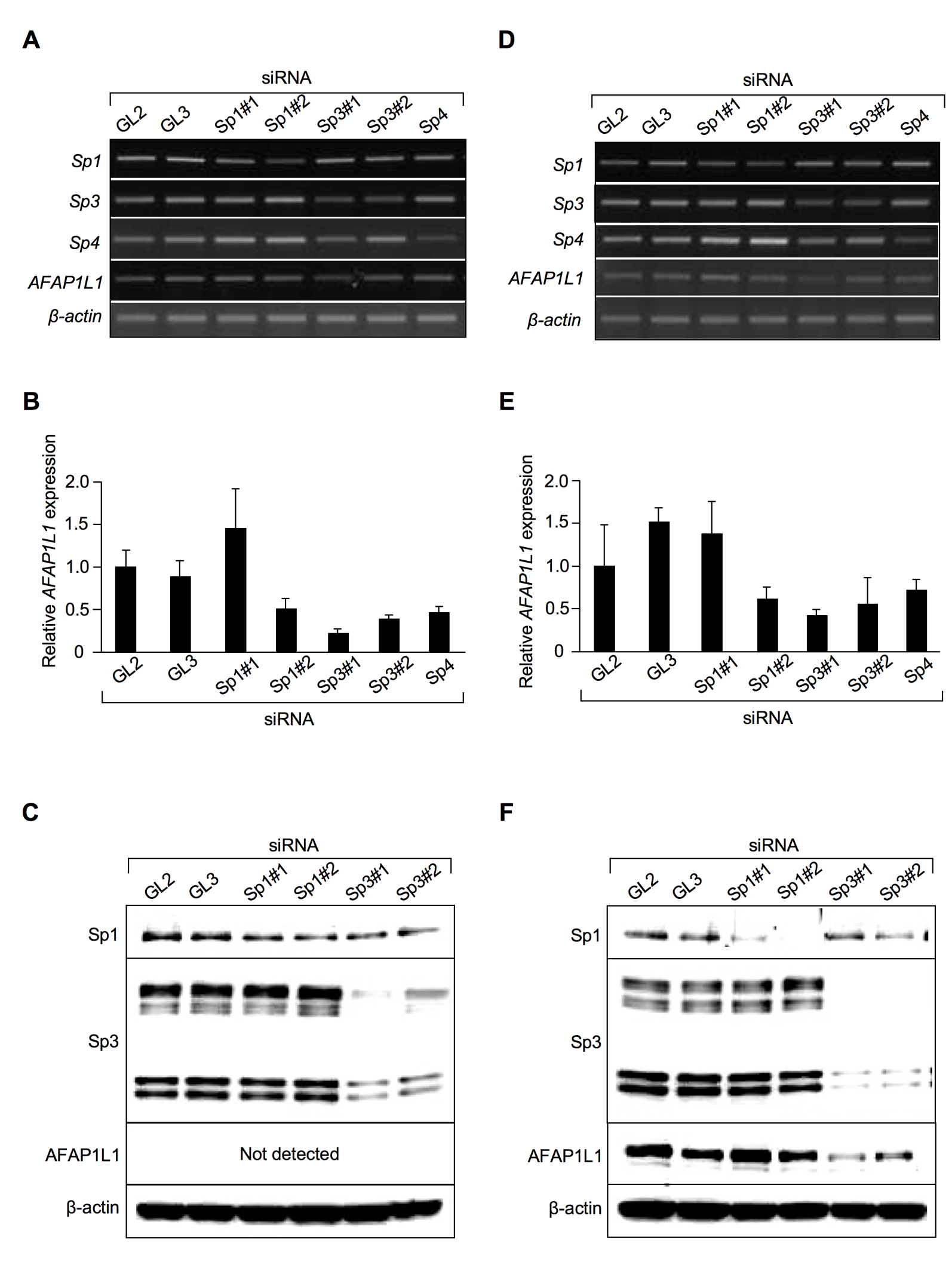

Supplement: Figure S8 — Down-regulation of AFAP1L1 expression by siRNA targeting the Sp3 gene in SYO-1 and MG63 cells. (A) and (D) The specificity of siRNA. SYO-1 (A) and MG63 (D) cells were treated with siRNA targeting Sp1, Sp3, or Sp4 for 48 h, and the expression of these genes as well as the AFAP1L1 gene was analyzed by PCR. Two different siRNAs targeting the Sp1 and Sp3 genes were designed and used. β-actin was used as a control. (B) and (E) Down-regulation of AFAP1L1 expression by siRNA targeting the Sp3 gene at the mRNA level. SYO-1 (B) and MG63 (E) cells were treated with siRNAs targeting each gene for 48 h and the expression of AFAP1L1 was analyzed by qPCR and indicated as fold changes relative to that in untreated cells. (C) and (F) Down-regulation of AFAP1L1 expression by siRNA targeting the Sp3 gene at the protein level. SYO-1 (C) and MG63 (F) cells were treated with siRNAs targeting each gene for 72 h and proteins were extracted and used for Western blotting. β-actin was used as a control. (TIF) [file pone.0049709.s008.tif]

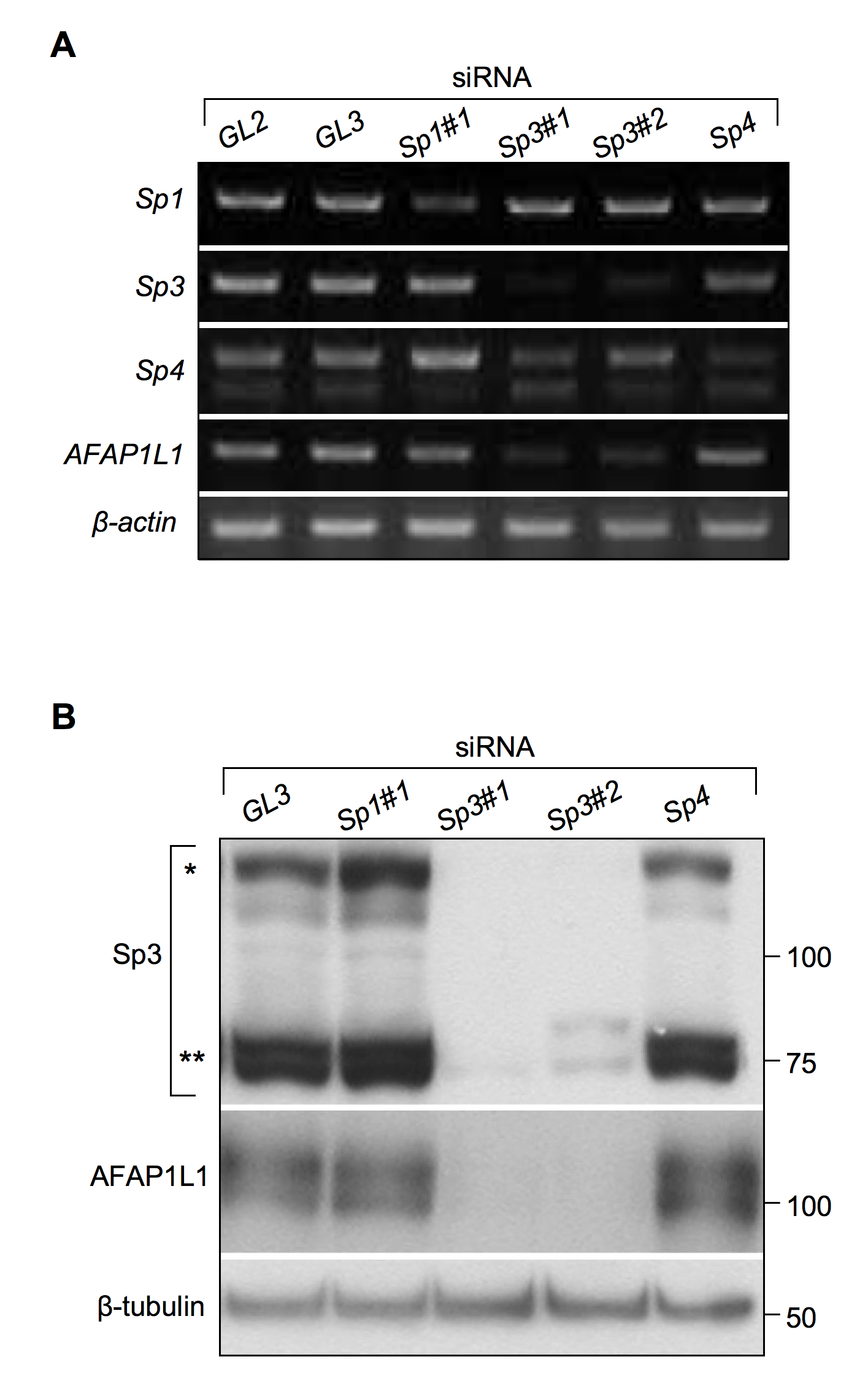

Supplement: Figure S9 — Down-regulation of Sp3 expression causes down-regulation of AFAP1L1 expression in prostate cancer cells. (A) The specificity of siRNA. PC-3 cells were treated with siRNA targeting Sp1, Sp3, or Sp4 for 48 h, and the expression of these genes as well as the AFAP1L1 gene was analyzed by PCR. Two different siRNAs targeting the Sp1 or Sp3 gene were designed and used. β-actin was used as a control. (B) Down-regulation of AFAP1L1 expression by siRNA targeting the Sp3 gene at the protein level. PC-3 cells were treated with siRNA targeting each gene for 72 h and proteins were extracted and used for Western blotting. β-tubulin was used as a control. Single and double asterisks indicate the long and short forms of Sp3, respectively. (TIF) [file pone.0049709.s009.tif]
